# Revisiting immunosurveillance and immunostimulation: Implications for cancer immunotherapy

**DOI:** 10.1186/1479-5876-3-8

**Published:** 2005-02-08

**Authors:** Christine V Ichim

**Affiliations:** 1 Department of Medical Biophysics, University of Toronto, Toronto, Ontario, Canada; 2 Department of Molecular and Cellular Biology, Sunnybrook and Women's College Health Sciences Centre, University of Toronto, 2075 Bayview Avenue, Toronto, Ontario, M4N 3M5 Canada; 3 MedVax Pharma Corp, 126 Madison Avenue South, Kitchener, ON N2G 3M6, Canada

## Abstract

Experimental and clinical experience demonstrates that the resolution of a pathogenic challenge depends not only on the presence or absence of an immune reaction, but also on the initiation of the proper type of immune reaction. The initiation of a non-protective type of immune reaction will not only result in a lack of protection, but may also exacerbate the underlying condition. For example, in cancer, constituents of the immune system have been shown to augment tumor proliferation, angiogenesis, and metastases. This review discusses the duality of the role of the immune system in cancer, from the theories of immunosurveillance and immunostimulation to current studies, which illustrate that the immune system has both a protective role and a tumor-promoting role in neoplasia. The potential of using chemotherapy to inhibit a tumor-promoting immune reaction is also discussed.

## 

*If only it were all so simple. If only there were evil people somewhere insidiously committing evil deeds, and it were necessary only to separate them from the rest of us and destroy them. But the line dividing good and evil cuts through the heart of every human being, and who is willing to destroy his own heart? *Alexander Solzhenitsyn, The Gulag Archipelago

The notion that the immune system may be manipulated into recognizing and eradicating neoplasia is not new. Heroic efforts to develop a cancer vaccine can be traced as far back as 1777 when the surgeon to the Duke of Kent injected himself with malignant tissue as a prophylaxis against development of cancer. In 1808, another attempt was made to develop a cancer vaccine by the doctor to Louis XVII who inoculated himself with breast cancer in hope of reversing a soft-tissue sarcoma, although no therapeutic effect was observed. However, it was not until 1891 that the first report of successful immunotherapy was published by William Coley, a clinician at the Memorial Sloan Kettering Cancer Institute in New York. Using heat-killed endotoxin-containing bacteria (streptococci and *Serratia marcescens*), Coley was able to achieve a cure rate of 10% in soft-tissue sarcoma [1,2]. Nevertheless, despite the numerous attempts over the past centuries to use the immune system in the eradication of cancer, the success rate of cellular immunotherapy remains abysmally low.

In light of the successes in the development of vaccines targeting pathogenic agents, this review suggests that lessons learned from the immunology of infectious disease may be applicable to the treatment of neoplasia. The immunology of infectious disease teaches that the clearance of a pathogenic challenge requires the initiation of an immune response of the appropriate quality and quantity. Clinical experience demonstrates the perils of an inappropriate immune reaction. Pathogens may be of an intracellular (viral, some bacterial strains) or extracellular (bacterial, parasitic) nature; accordingly, the specific immune response is bifurcated into a cell-mediated branch, which offers protection against intracellular pathogens, and an antibody-mediated branch, which offers protection against extracellular pathogens. Following a challenge with *Mycobacterium leprae*, the leprosy inducing bacterium, a cell-mediated immune response will result in protection whereas an antibody-mediated immune response will result in cachexia and disease progression [3]. The ability of the immune system to act as a double-edged sword implies that in any given condition the initiation of an immune response may result in either protection or destruction of healthy tissue. An appreciation of the duality of the immune reaction is imperative in the design of immunotherapeutic approaches that attempt to attain a therapeutic benefit through the manipulation of the immune system.

Two distinct theories aim to define the role of the immune system in cancer. The theory of immunosurveillance postulates that the role of the immune response in cancer is one of protection – the organism is patrolled for incipient tumor cells by the effector cells of the immune system. In contrast, the theory of immunostimulation postulates that, while in experimental systems highly immunogenic tumors may be eradicated by the immune response, the role of the immune response in spontaneous neoplasia is not one of protection, but rather of tumor promotion. This review gives a historical overview of these theories and highlights recent data supporting the validity of both immunosurveillance and immunostimulation. To explain the conflicting roles of the immune response in neoplasia it must be noted that the immune system is not a single entity, but a complex system of constituents. While these theories have historically been considered to be mutually exclusive, it is proposed that these theories describe the activation of different constituents of the immune system and hence illustrate that an appropriate immune reaction will result in protection whereas an inappropriate immune reaction will result in tumor promotion.

## The theory of immunosurveillance

### The rise of the theory of immunosurveillance

As early as 1909 Paul Ehrlich postulated that cancer occurs spontaneously *in vivo *and that the immune system is able to both recognize and protect against it [4]. In the late 1950s Lewis Thomas [5] introduced the theory of immunosurveillance, which was subsequently developed by Sir MacFarlane Burnet [6]. The theory postulates that effector cells of the immune system actively patrol the body to identify and eradicate incipient tumor cells. Following the identification of T cells in the 1970s, these became the effector cells postulated to mediate immunosurveillance.

The concept of immunosurveillance initially had much intellectual appeal. First, it could explain clinical observations of spontaneous remission. Second, the most potent chemical carcinogens, dimethlybenzanthracene [7], urethan [8] and other polycyclic hydrocarbons [9], are also powerful immunosuppressors, their suppressive effects being apparent even after a single exposure [7]. Third, specific immune responses had been observed in the transplantation of chemically induced tumors. Finally, the theory of immunosurveillance was developed at a time when the only known function of T cells was to reject foreign grafts; thus, there was a clear need to further determine the biological function of these cells.

### Discrepancies in the theory of immunosurveillance

A number of discrepancies were noted in the theory of immunosurveillance as initially described by Thomas and Burnet, where T-cells function as the effector cells mediating immunosurveillance. First, T-cell deficient (athymic nude) mice did not develop significantly more cancers than control mice [10] Second, a corollary of the theory of immunosurveillance is that patients who lack an immune response would have an increased incidence of neoplasia. While this has been reported in immunosuppressed patients who have undergone renal transplant, most of these tumors (as many as 60%) were reported to be hematological malignancies or neoplasms with viral etiology. It would be reasonable to expect the immune system to protect against viral carcinogenesis, as the protective role of the immune system against viral pathogens is well established. One interpretation of these results is that the predisposition toward hematological malignancy is due to the viral etiology of certain lymphomas (which are thought to be caused by the Epstein-Barr virus). The only non-lymphoid tumors that increase significantly upon immunosuppression are non-melanoma skin cancers, cervical carcinoma and Kaposi's sarcoma (the latter two having a viral etiology, specifically the human papilloma virus and human herpes virus 8, respectively). In these early studies, the incidence of most tumor types was not increased by immunosuppression [11]. In fact, the incidence of mammary carcinomas actually decreases in immunosuppressed individuals [12]. A third discrepancy is that diseases such as leprosy, sarcoidosis and uremia, which are characterized by immunosuppression, are not accompanied by an increased incidence of tumors [13,14]. Finally, the theory of immunosurveillance predicts that sites that are excluded from the immune system – that is, sites of immunological privilege such as the anterior chamber of the eye and the brain – should have an increased incidence of cancer. However, this is not the case.

Furthermore, several studies suggest that incipient tumors may not be immunogenic. A correlation between the dose of carcinogen administrated and the immunogenicity of the subsequent tumor has been reported in mouse models [15-17]. Since human tumors most likely occur as a result of low exposure to carcinogens, incipient tumors would be expected to be poorly immunogenic. In addition, since incipient tumors would contain few cells, the question arises of whether the antigenic load would be sufficient to induce a response [18]. This phenomenon may be observed in the immunology of infectious diseases where naïve C57Bl/6 mice challenged with *Taenia taeniaeformis *will reject a large inoculum of the cestode larva yet will succumb to infection by minute inoculations [19]. Finally, it is only in recently years that the tumor specific- and not tumor associated antigens have been identified [20].

### The revivification of immunosurveillance

Over the past two decades, data have emerged suggesting that constituents of the immune system such as natural killer (NK) cells and cytokine networks may be able to guard against cancer. Furthermore, recent studies of the incidence of neoplasia in immunosuppressed patients after organ transplantation, in contrast to earlier studies, reported that these patients are more susceptible to a wide range of cancers, including epithelial cancers [21,22].

#### Natural killer cells

Several studies suggest that NK cells are able to protect against tumors. Beige, natural-killer-cell defective, mice have an increased incidence of spontaneous tumors [23] and cancer metastases [24-28]. This is consistent with the pattern seen in patients with Chediak Higashi syndrome, an autosomal recessive disorder characterised by abnormal NK cytotoxic function. These patients also have a 200-fold increased risk of developing malignancy [29]. Furthermore, several studies show that cancer cells secrete soluble factors that suppress NK cells *in vitro *[30-32] and that patients with a variety of tumor types have suppressed NK cell activity [33-38]. NK cell activity is also a positive prognostic indicator in several tumor types [39-43]. These data suggest that NK cells are involved, directly or indirectly, in the surveillance of incipient tumors and micrometastases. This theory is further substantiated by data showing that oncogene-transfected fibroblasts can be selectively lysed by NK cells while sparing untransfected controls [44].

The precise mechanism though which NK cells mediated immunosurveillance is not yet understood. It is likely that the role of NK cells, in addition to their direct cytotoxic effects, is to activate other cells of the immune system through providing cytokine support [45]. Mature NK cells do not produce T-helper 2 (Th2) cytokines, but rather the T-helper1 (Th1) cytokines tumor necrosis factor (TNF)-α, interferon (IFN)-γ and GM-CSF [46]. In fact, secretion of IFN-γ by NK cells can influence the development of a Th1 type immune response both against pathogenic agents and against MCA-induced tumors [47].

#### Cytokine networks

The initiation of an immune response requires cytokine support provided by T-helper cells. The nuance of cytokines produced by these cells determines the type of immune response initiated. The initiation of a cell-mediated immune response requires the support of Th1 cytokines, while the initiation of an antibody-mediated immune response requires the support of Th2 cytokines. T-helper cells that have differentiated into Th1 cells secrete IFN-γ and to a lesser extent interleukin (IL)-2 and IL-12, whereas Th2 cells secrete IL-10, IL-4 and to a lesser extent IL-5.

The cell-mediated immune response is generally regarded as possessing tumor-inhibitory activities both clinically and in animal models [48-51]. Accordingly, a number of studies suggest that the expression of Th1 cytokines is associated with a favorable clinical outcome, while the expression of Th2 cytokines is associated with an unfavorable clinical outcome. In renal-cell carcinoma, the presence of an IL-4 receptor polymorphism that resulted in an increase in IL-4 signaling and an increased likelihood of a Th2 response was an independent indicator of adverse prognosis [52]. In other studies of patients with renal cancer, an elevated level of IL-10 was an adverse prognostic indicator [53], while the serum levels of IFN-γ were negatively correlated with tumor mass [54]. In malignant melanoma, patients that had an early relapse had lower serum levels of IL-2 and IL-12. Furthermore, decreases in the serum concentrations of IL-2 and IL-12 and increases in IL-10 were observed at least one month before relapse [55]. In B-cell diffuse large cell lymphoma, patients who achieved complete remission had a higher ratio of Th1 to Th2 cells [56]. Finally, in a range of advanced solid cancers, the serum IL-10 level was an independent prognostic indicator of overall survival and time to treatment failure [57]. These data suggest that the Th1-Th2 paradigm is relevant to cancer, although it should be noted that the Th1/Th2 paradigm is a generalization and that these data show a correlation, and as such are not evidence of a causal relationship. Since many tumors secrete IL-10, it is possible, for example, that the relationship between expression of IL-10 and tumor prognosis reflects the poor prognosis of patients with a larger tumor burden. This is plausible given that the role of IL-10 in tumor immunology is complex (reviewed in [58]). IL-10 appears to be a pleotropic cytokine whose reported effect is a function of the nuances of the experimental system. While several reports suggest that IL-10 is immunosuppressive, several groups cite IL-10 to have stimulatory effects on T cells and NK cells [59]. In addition, IL-10 transfected into murine carcinoma models decreases tumorigenicity and sensitizes tumor cells to immune mediated lysis by either NK cells or NK cells and cytotoxic T-cells [60-62]. Clinical data further corroborates a protective role for IL-10 in tumor immunology by showing that metastatic melanoma patients who responded to an immunotherapeutic regimen had tumors with significantly higher expression of IL-10 mRNA as compared to patients that did not respond [63,64].

Furthermore, tumors have a number of specific and non-specific ways of evading a Th1 response. Tumors secrete a number of agents, including transforming growth factor (TGF)-β, IL-10 and prostaglandin E-2, which have been shown to promote a Th2 immune response while suppressing the Th1 immune response. Indeed, it has been shown that the cytokine networks of some cancer patients are skewed toward Th2 [55,65,66]. That is, these patients exhibit enhanced expression of Th2 cytokines or decreased expression of Th1 cytokines systemically or in the local tumor microenvironment. The observation that tumors have developed numerous methods of evading the Th-1 response is consistent with the notion of immunosurveillance. Nowell's clonal-evolution hypothesis postulates that cancer is a Darwinian process: mutations that provide a growth or survival advantage will be selected for in the population [67]. The fact that malignancies have many ways of evading the Th-1 response suggests that the ability to evade this response confers a survival advantage on malignant cells, which further suggests that the Th-1 response poses a threat to the neoplasm.

#### Interferon-γ

The Th-1 cytokine IFN-γ has both direct and indirect antitumor properties. A series of experiments have demonstrated the importance of IFN-γ in eradicating incipient tumors by showing an increase in the efficacy of carcinogenesis in the absence of IFN-γ. These experiments were conducted either with neutralizing antibodies to IFN-γ or in animal models deficient in IFN-γ, the IFN-γ receptor or downstream signaling mechanisms of IFN γ, specifically, the signal transducer and activator of transcription-1 protein. In all of these models, an increase in the incidence of MCA-induced tumors and a decrease in the latency period of the tumors were observed [68-71]. Furthermore, an increase in the number of spontaneous lymphomas and lung tumors was observed in IFN-γ deficient mice compared with genetically matched wild-type controls [72].

IFN-γ may inhibit tumor growth by affecting proliferation, apoptosis and angiogenesis. IFN-γ has been shown to have a direct anti-proliferative effect on certain tumor models [73-75]. It is thought that this effect is mediated through p21 (WAF1/CIP1) and p27 (kip1) as IFN-γ activates these tumor suppressors [76,77]. Furthermore, IFN-γ has been reported to modulate apoptosis in certain models [78,79] by inducing expression of caspase 1 and Fas/FasL [80,81]. With respect to angiogenesis, IFN-γ induces expression of three angiostatic non-ELR (non-Glu-Leu-Arg) chemokines: interferon-gamma-inducible protein 10 (IP-10), monokine induced by gamma interferon and interferon-inducible T-cell alpha chemoattractant [82-84]. The effects of these chemokines on angiogenesis will be discussed.

IFN-γ may also have indirect antitumor effects by stimulating an effective antitumor immune response. In addition to influencing the Th1-Th2 cytokine balance, IFN-γ can activate cytotoxic macrophages, NK cells and NK T cells [85].

## The theory of immunostimulation

The proof of the principle that an inappropriate type of immune response will enhance tumor growth was demonstrated as early as 1907 by Flexner and Jobling, who showed that injection of dead autologous tumor cells enhanced the growth of pre-existing tumors [86]. In general, Th2-driven antibody responses to tumors are non-protective and may contribute to tumor progression by inhibiting the Th1 cell-mediated immune response [87]. However, the notion that the antibody-mediated immune response may be detrimental in cancer was suggested long before Mossman and Coffman demonstrated the Th1-Th2 paradigm in 1986 [88]. In the 1950s Kaliss popularized the term "immunological enhancement" to describe the enhancement of tumor growth by non-cytotoxic antibodies [89]. It was theorized that these antibodies bind to tumor cells, masking their epitopes and thus preventing a cell-mediated immune response, although this has never been demonstrated experimentally.

In 1972, Richmond Prehn formulated the theory of immunostimulation of tumor growth [90]. This theory states that, in contrast to the strong immune response generated by transplantable tumors, a quantitatively mild immune response, such as that generated by spontaneous tumors, is stimulatory to the growth of neoplasia. Several experimental observations support the hypothesis that such a weak immune response to cancer may stimulate tumor growth. The co-injection of lymphocytes (spleen cells) from syngeneic mice that had been growing tumors for 10–20 days with tumor cells from MCA-induced sarcomas into thymectomized irradiated syngeneic mice at a range of doses accelerated tumor growth when the ratio of lymphocytes to tumor cells was low [90]. However, when the ratio of lymphocytes to tumor cells was high, lymphocytes from specifically immunized mice inhibited growth compared with naïve lymphocytes that continued to augment tumor growth. This suggests the existence of a biphasic dose response whereas a "weak" immune response results in stimulation of tumor growth while a strong immune response results in protection.

This premise is also demonstrated in another study where MCA induction of tumors occurred more rapidly in irradiated thymectomized mice that had received increasing doses of lymphocytes (spleen cells) to partially restore their immune system than in mice that had either fully restored or unrestored immune systems [91]. Comparable results have been observed in mice that have had their immune systems compromised to various degrees by irradiation [92] and in T-cell deficient nude mice that have had their immune systems partially restored by the injection of various quantities of thymic or spleen cells [93]. These studies support the notion that a weak antitumor immune response will not confer protection and may exacerbate tumor growth.

Furthermore, in transplantation immunology, an immune response against a graft generally promotes graft rejection. However, rabbits that received skin grafts of similar but not identical major histocompatibility complex launched an immune response that resulted not in rejection but in enhanced growth of the tissue, resulting in hyperplasia [94]. This is consistent with the notion that a quantitatively "weak" immune response against a tumor may enhance tumor growth.

Collectively, these data support the essence of the theory of immunostimulation: specifically, that spontaneous tumors may not stimulate an appropriate immune response but rather stimulate non-protective immune responses that quantitatively are not adequate for tumor eradication. These resulting "weak" immune responses are not merely non-protective but actually facilitate tumor growth. Specifically, a "weak" immune response is a state in which immunological recognition of the tumor occurs but resolution is not achieved. As this theory was developed in an era when our understanding of the composition of the immune system was limited, the exact mechanism by which a "weak" immune response stimulates tumor growth has yet to be defined in the terminology of contemporary immunology. Recent data, however, suggest numerous mechanisms by which the immune system can facilitate tumor growth and progression.

### Inappropriate immune reactions exacerbate cancer

Based on the observation that a tumor is in a state of chronic inflammation, Dvorak compared cancer to a wound that never heals [95]. Numerous tumor types constitutively produce cytokines and chemokines; this results in the migration of leukocytes into tumors. Unfortunately, these immune infiltrates often do not offer protection but actually facilitate tumor growth. Tumor-infiltrating leukocytes can facilitate tumor progression by secreting growth factors, reactive oxygen and nitrogen species, proteases, prostaglandins and angiogenic growth factors. Reactive oxygen and nitrogen species may directly promote tumor progression by inducing DNA damage, and hence the acquisition of additional mutations. In addition, these cells may skew the cytokine milieu to favor the generation of a non-protective Th2 immune response. Mast cells and macrophages are examples of inflammatory cells that are often recruited to tumor sites through chemotactic gradients. The mechanisms by which mast cells, macrophages and chemokines themselves facilitate tumor growth and progression are discussed below.

#### Mast cells

Mast cells are leukocytes that contain inflammatory agents, such as proteases, histamine and heparin, and mediate hypersensitivity reactions. The possibility that mast cells are involved in cancer has been considered since the late 1800s. In 1877, Paul Ehrlich described a mast cell for the first time in his doctoral thesis, after identifying it using histological staining. In subsequent investigations, he and others observed that mast cells localize around tumor tissues [96]. Interestingly, mast cells were more likely to be localized in the periphery than in the centre of tumor sections. Many types of human tumors contain mast-cell infiltrates [97-100]. The accumulation of mast cells in neoplastic tissue results from the secretion of growth factors such as vascular endothelial growth factor (VEGF) [101], epidermal growth factor [102], basic fibroblast growth factor (bFGF) [103,104], platelet derived growth factor [101] and stem cell factor (SCF) [105] by the tumor. These cytokines act as chemotactic agents to induce the migration of mast cells.

A number of studies show a correlation between mast-cell infiltration and tumor progression. For example, co-injection of mast cells with an inoculum of rat sarcoma tumors results in enhanced tumor growth, while pharmacologically decreasing the quantity of mast cells slows tumor growth [106]. Furthermore, genetic evidence supports the role of mast cells in tumor growth and progression. SCF is important for mast-cell development, proliferation, migration and degranulation. W/W^v ^mice (which express a mutation in the SCF receptor) do not develop functional mast cells. In W/W^v ^mice, tumors do not metastasize as readily and they vascularize at a slower rate than in wild-type mice. Tumor metastasis and vascularization return to wild-type levels following restoration of mast cells [107]. Furthermore, administering antisense targeting SCF in a rat mammary-tumor model results in a decrease in mast-cell degranulation, microvascular density and tumor growth [108]. These studies suggest that the presence of mast cells in tumors is not indicative of a protective immune reaction, as these cells actually facilitate tumor progression.

The mechanisms by which mast cells facilitate tumor progression appear to involve the factors contained within mast-cell granules. Experimental evidence suggests that degranulation is critical in the ability of mast cells to enhance tumor growth. Inhibition of mast-cell degranulation using disodium cromoglycate impedes tumor growth [109]. One of the functions of degranulation in tumor growth may be to facilitate angiogenesis. Addition of mast cells or isolated mast-cell granules induces vascularization in the chorioallantoic membrane model, while no effect of adding previously degranulated mast cells was observed [110]. This effect appears to be partially mediated through bFGF and VEGF, as addition of anti-bFGF and anti-VEGF antibodies significantly decreased the degree of vascularization. Indeed, mast cells secrete a number of growth factors that regulate angiogenesis and endothelial-cell survival, including TNF-α [111], IL-8 [111], bFGF [112] and VEGF [113,114]. Moreover, mast-cell granules contain the proteolytic enzyme tryptase, while some mast-cell subsets also contain chymase. These enzymes have both direct and indirect effects on the integrity of the extracellular matrix (ECM). Directly, these enzymes induce degradation of ECM components [115]. Indirectly, they can induce degradation of the ECM by activating latent forms of matrix metalloproteinases [116]. Furthermore, it has been reported that these proteinases may induce proliferation of vascular endothelial cells [117].

As is often the case in networks, activation of one component induces the activation or suppression of interacting components. Activated mast cells secrete a number of cytokines that induce the chemotactic migration of macrophages into the tumor, including IL-1, IL-6, TNF-α, IL-8, monocyte chemotactic protein-1, macrophage inflammatory protein-1α and macrophage inflammatory protein-1β [118,119]. The effect of macrophages on tumor growth and progression is discussed in the following section. In addition, activation of mast cells induces the synthesis of the cylooxygenase enzyme, which is involved in the metabolism of arachidonic acid [120-122] and prostaglandins. Pharmacological inhibition of cylooxygenase significantly impedes metastasis in several animal models [123-125].

#### Macrophages

Macrophage infiltration of tumor sites has been observed in a range of tumor types. Monocytes are recruited to the tumor by constitutive tumor-cell expression of chemoattractant cytokines. In fact, the level of expression of monocyte chemotactic protein-1, macrophage colony stimulating factor (M-CSF) and VEGF by tumor cells correlates well with the extent of macrophage infiltration [126,127]. Within the tumor microenvironment, monocytes differentiate into macrophages. The role of the macrophage in the tumor microenvironment appears to be specific to the tumor type. In breast [128], cervical [129] and bladder [130] cancers macrophage infiltration is an adverse prognostic indicator. However, in prostate [131,132], lung [133,134] and brain [135,136] cancers the prognostic significance of tumor associated macrophages, TAMs, depends on the method of assessing macrophage infiltration, the endpoints of the study and the specifics of the patient cohort. This discrepancy highlights the chameleon-like nature of the macrophage. Depending on their microenvironment, macrophages may either exhibit antitumor cytotoxic activity or facilitate tumor growth and progression while reinforcing a Th2 biased immune response [137].

Following exposure to IFN-γ or bacterial lipopolysaccharide, macrophages can exhibit direct or indirect tumor cytotoxicity. Macrophages may participate in antibody-dependent cellular cytotoxicity responses if an IgG2a antibody binds to a tumor surface antigen. In such a scenario, the macrophage would bind to the Fc portion of the antibody, releasing cytotoxic mediators such as proteinases and TNF-α. Macrophages may also mediate cytotoxicity independently of antibodies through the secretion of reactive oxygen and nitrogen species, proteinases and TNF-α. Furthermore, macrophages of this "cytotoxic" phenotype affect the cytokine profile of the microenvironment because they secrete IL-12. IL-12 favors the differentiation of naïve T-helper cells to Th1 cells, which will subsequently secrete IFN-γ and TNF-β. The role of these cytokines in the promotion of a cell-mediated immune response has already been discussed. Finally, cytotoxic macrophages produce matrix metalloproteinase-12 [138]. Matrix metalloproteinase-12 has been shown to convert plasminogen to angiostatin [139]. Angiostatin is important in the inhibition of angiogenesis [140].

In contrast to macrophages that are activated by classical mechanisms and are capable of cytotoxic activity, TAMs, for the most part, do not exhibit cytotoxic activity, are Th1 immunosuppressive and, in fact, facilitate tumor growth, vascularization and metastasis. Tumors secrete a number of cytokines including IL-4, IL-10, TGF-β, prostaglandin E-2 and VEGF. These factors modulate the macrophage phenotype, changing them from cytotoxic macrophages to suppressive macrophages [141-143]. Furthermore, TAMs appear to have functional defects; for example, in contrast to other macrophages, TAMs are poor antigen-presenting cells [141] and have reduced cytotoxicity owing to impaired production of TNF-α [144] and nitric oxide [145]. However, these impairments in the function of TAMs are consistent with macrophages that are not activated rather than defective.

A number of studies suggest that TAMs facilitate tumor growth, vascularization and metastases. As previously stated, numerous studies have shown the presence of TAMs to be an adverse prognostic indicator. In addition, genetic evidence suggests that macrophages are important in vascularization and metastasis of experimental tumors. To assess the role of macrophages in carcinogenesis, the M-CSF deficient osteoporotic (op/op) mouse was crossed with the polyoma virus middle T transgenic mouse, which develops spontaneous mammary tumors. While no differences were observed in the early stages of tumor growth, the resulting offspring of these tumor-bearing mice had a reduced rate of progression to invasive carcinoma and contained fewer pulmonary metastases than control mice [146]. Furthermore, induced M-CSF expression in the mammary tissue of these mice resulted in restored metastatic spread of experimental tumors. In addition, transplantation of the Lewis lung carcinoma in the op/op mouse resulted in a decrease in its growth and vascularization compared with wild-type littermates [147]. This attenuation could be reversed by administration of M-CSF. These data suggest that macrophage infiltration is important in angiogenesis and tumor progression.

Interestingly, TAMs are not found ubiquitously within tumor tissue. Data suggest that TAMs localize to poorly vascularized regions – that is, regions characterized by hypoxia, low pH and tissue necrosis [127,148-150]. In response to hypoxia, macrophages express a number of hypoxia-regulated gene-products, including VEGF, hypoxia inducible factor 1α and hypoxia inducible factor 2α [149,151,152]. Furthermore, hypoxic conditions impair the chemotactic migration of macrophages [150,153]. This suggests that macrophages could be detained in regions of low oxygen tension. While the precise nature of the relationship between macrophages and hypoxia requires further investigation (macrophages are phagocytic cells, so their physiological role may well be to engulf necrotic tissues), it is possible that macrophage recruitment to these sites facilitates angiogenesis.

One mechanism by which TAMs may augment angiogenesis is through the production of a plethora of cytokines and proteinases. As mentioned above, hypoxia induces production of VEGF. The angiogenic properties of VEGF have been extensively discussed by others [154]. In addition to VEGF, the repertoire of cytokines produced by TAMs includes granulocyte macrophage colony-stimulating factor, TGF-α, TGF-β, IL-1, IL-6, IL-8 and prostaglandin E-2. These cytokines are involved in the regulation of angiogenesis. Furthermore, TAMs secrete urokinase-type plasminogen activator and matrix metalloproteinase-9 [155,156]. These proteinases are involved in the induction of angiogenesis and metastasis through degradation of the ECM.

Finally, TAMs have numerous indirect immunological effects. TAMs produce copious amounts of IL-10 but low amounts of IL-12 [157]. This shift in the cytokine profile reinforces the Th2 imbalance that is often present in cancer models. More significantly, IL-10 prevents T-cell activation by inducing a state of anergy (a state of T-cell non-responsiveness associated with tolerance induction). IL-10 can induce anergy both in T cells that have been activated in its presence and in T-cells activated by antigen-presenting cells that were previously exposed to IL-10 [158]. These data suggest that TAMs may also facilitate tumor growth by inducing tolerance to the tumor and hence suppressing the antitumor immune response.

#### Chemokines

Chemokines are a subclass of cytokines that direct the migration of leukocytes to sites of inflammation. It has recently been observed that tumors express chemokine receptors [159-161]. Thus, it should not come as a surprise that, aside from their role in mediating the recruitment of tumor-infiltrating leukocytes to tumor sites, chemokines may also affect neoplastic proliferation, neovascularization and metastasis. Specific chemokines, such as growth-related oncogene (GRO)-α, GRO-β, GRO-γ and IL-8, directly induce the proliferation of melanoma cells [162], while ligands of the chemokine receptor CXCR-2, such as IL-8, induce proliferation of lung [163,164], ovarian [165], pancreatic [166] and head and neck [167] tumors. Chemokines may both augment and inhibit angiogenesis. In general, chemokines with an ELR motif promote angiogenesis and facilitate the chemotactic migration of endothelial cells [168]. Nevertheless, IFN-γ induces the expression of three non-ELR chemokines that have been reported to have angiostatic properties. For example, expression of IP-10 is negatively correlated with human lung-cancer tumor growth [169], while administering recombinant IP-10 slows tumor growth [170]. Furthermore, administration of IL-12 inhibits bFGF-mediated *in vivo *neovascularization of matrigel [171]. The angiostatic effects of IL-12 appear to be mediated through the induction of IP-10 and of monokine induced by gamma interferon, as neutralizing antibodies to these chemokines negated the angiostatic effects of IL-12 [172,173]. Finally, in some systems IL-8 activates the transcription of matrix metalloproteinase and is associated with an increased degree of invasion and metastasis. Furthermore, chemokines may be involved in the attraction of circulating cancer cells to sites of metastasis [174-177].

## Strategic research directions

Based on our knowledge of tumor immune evasion and immune contribution to growth of tumors, several therapeutic implications arise including: a) Use of adjuvant therapies to decrease tumor immune suppression while vaccination, b) Extracorporeal elimination of immune suppressive molecules; and c) Gene silencing of tumors to generate immunity.

The armamentarium of clinically useful drugs for inhibition of tumor immunity is rapidly growing. The challenge is to identify drugs that intrinsically possess antitumor activity, while at the same time can reverse immune stimulation. One promising candidate that fits these criteria is the clinically used anti-VEGF antibody bevacizumab (Avastin). Currently approved by the FDA for treatment of advanced colon cancer patients, this antibody is believed to mediate its effects primarily by inhibition of angiogenesis [178]. From the tumor immunotherapy perspective, VEGF plays an important role for tumor suppression of immune responses. In fact, administration of anti-VEGF antibody increases efficacy of immunotherapy in mouse models [179]. Mechanisms by which VEGF inhibits antitumor immunity include suppression of NF-kB activation in DC resulting in an immature phenotype [180], as well as suppression of T cell activation [181]. Suppression of immune inhibitory molecules could also be accomplished by immunization. For example, the pregnancy associated molecule human chorionic gonadotropin (hCG) is associated with inhibition of Th1 generation *in vitro *as well as *in vivo *[182].

Vaccination with the carboxy-terminal peptide of hCG has been shown to break tolerance to this self-molecule and induce a marginal anticancer response [183]. Combining vaccination against immune suppressive molecules with vaccination against tumor-specific antigens will likely improve efficacy.

Another method of reversing tumor associated immune suppression would involve extracorporeal removal of inhibitory molecules. Although plasmapheresis techniques have been attempted with mediocre success [184], a novel and promising method involves ultrapheresis. Lentz et al described a pilot study of 16 metastatic patients in which the <100,000 kDa fraction was removed through membrane ultrapheresis, Six of the 16 patients had reduction of the sum of mean cross-sectional diameters of measureable lesions by 50% or more. Additionally, tumor infiltrating lymphocytes were observed in many of the lesions after 2 months of treatment [185]. Follow-up studies demonstrated that the immune suppressive component being removed through the ultrapheresis procedure was soluble TNF-R alpha [186]. Recent developments in hollow-fiber technologies allow for specific removal of plasma bourne components such as HIV gp120 and various toxins [187]. Applications of these techniques to immune therapy could yield valuable new approaches that are currently not studied.

The introduction of RNA interference (RNAi) as a potent method of gene-specific silencing has opened new territories for gene therapy. Successful use of RNAi for immune modulation was first reported by Hill et al [188]. Subsequently, it was demonstrated that silencing of the inhibitory cytokine IL-10 led to the generation of dendritic cells with potent Th1 priming abilities [189]. More recently, siRNA modified DC were used for induction of antitumor immunity [190]. The fact that naked siRNA can be directly endocytosed by target cells [191], or administered using polyethylenimine-complexe [192], suggests the possibility of direct intratumoral injection of siRNA targeting immune suppressive molecules. Additionally, tumor-targeting immunoliposomes could be used for systemic delivery of siRNA into cancer cells. Suitable targets would include the wide variety of immune suppressive molecules mentioned above.

## Conclusions

The debate on the role of the immune system in cancer has been one of the most controversial areas of science with opinions vacillating from optimistic highs to skeptic nadirs on a cyclical basis. These oscillations in opinion that are reflective of the relative progress in the immunotherapy of cancer, can be explained by the complexity of the relationship of the immune system and cancer – an interaction that can result in anti-tumor protection, tumor promotion, or in no net effect. The apparent schizoid role of the immune system in cancer may in fact be a variation on a theme borrowed from the immunology of infectious disease. That is, the mere generation of an immune response is not sufficient to attain protection against a pathogen: it is only an immune response of the proper type and the proper magnitude that will result in protection. An immune response against an agent that is not of the proper type and magnitude will be deleterious to the host.

A proper type of immune response against cancer will result in protection. Evidence exists that in set conditions components of the immune system can recognize and eradicate incipient cancer cells. It is likely that some sort of immune surveillance exists in the healthy individual – though the exact mechanisms have yet to be elucidated. The genetic evidence discussed herein suggests that NK cells and IFN-γ are likely to be involved in this protection.

Nevertheless, the clinical presentation of cancer suggests that neoplasia can evade these putative mechanisms of immunosurveillance. This further suggests that the ensuing immune response is not protective, or not adequately protective, to control the nascent tumor. However, this lack of a protective response is not the whole extent of the role of the immune system in cancer. Several examples have been presented in which the immune response actually facilitates tumor growth, neovascularization and progression. These observations suggest a mechanism that may explain the theory of immunostimulation, though undoubtedly, many other mechanisms may exist.

This duality of the immune system in cancer arises in part because of the decentralized nature of the immune system. The non-protective immune reaction that arises resembles a bureaucratic organization in which a lack of orchestration between departments results in redundancy and counter-productivity: the individual entities that constitute the immune system react to the presence of the tumor yet they are not orchestrated to achieve the end point of tumor eradication.

Secondly, in advanced tumors, the duality of the immune system in cancer may also arise from the bilateral nature of the interaction between the tumor and the immune mechanisms of the host. Not only can the immune system affect the tumor, but the tumor may also affect the immune system. The skewed cytokine milieu of a tumor, which results in the recruitment of inflammatory cells through the secretion of chemokines and growth factors and which further prevents the cytotoxic activation of these cells, is an example of how the tumor influences the immune system. This effect of the tumor on the immune system may be explained by the theory of immunoediting, which describes the iterative process of selection for cells that can evade the natural immunosurveillance mechanisms [193]. This selection process may generate tumors that not only escape detection by the immune system, but that have actually generated mechanisms of depressing those branches of the immune response that would offer anti-tumor protection and/or enhancing those branches that would elicit tumor promotion.

Akin to the successful development of vaccines against infectious agents, the development of effective immunotherapy against cancer will require recognition of the duality of the immune response in cancer, and the stimulation of the proper type of immune response. The challenges ahead, especially in late-stage patients who have undergone immunoediting, will be to overcome the tendencies of the tumor to elicit an inappropriate immune response, one used by the tumor to promote its own growth. It may be the case that in subsets of patients the sort of immunotherapy that is desirable is not that which stimulates the immune system but that which suppresses an inappropriate, tumor promoting, immune reaction [194,195]. Chemotherapy, may in fact be an example of 'immunotherapy'.

Chemotherapy both directly and indirectly affects the immune system; however, given the multiplicity of the functions of the immune system in cancer, the net effect of this treatment modality remains to be defined. While the direct effect of chemotherapy may be myelosuppression, this may not be an undesirable consequence given that cells of the myeloid lineage, such as mast cells and macrophages, often promote the growth, vascularization and invasion of tumors. Furthermore, myelosuppression may actually stimulate a protective, Th1-type, cell mediated immune response because mast cells and macrophages secrete factors that promote a Th2 type of immune response.

"If only it were all so simple." If only the immune system were all good and it were necessary only to stimulate it to achieve the eradication of tumors. But the potential for good and evil is entwined within the heart of the immune reaction, and who is willing to destroy their own heart?
